# Exocytosis of polyubiquitinated proteins in bortezomib-resistant leukemia cells: a role for MARCKS in acquired resistance to proteasome inhibitors

**DOI:** 10.18632/oncotarget.11340

**Published:** 2016-08-17

**Authors:** Niels E. Franke, Gertjan L. Kaspers, Yehuda G. Assaraf, Johan van Meerloo, Denise Niewerth, Floortje L. Kessler, Pino J. Poddighe, Jeroen Kole, Serge J. Smeets, Bauke Ylstra, Chonglei Bi, Wee Joo Chng, Terzah M. Horton, Rene X. Menezes, Renée J.P. Musters, Sonja Zweegman, Gerrit Jansen, Jacqueline Cloos

**Affiliations:** ^1^ Department of Pediatric Oncology/Hematology, VU University Medical Center, Amsterdam, The Netherlands; ^2^ The Fred Wyszkowski Cancer Research Laboratory, Technion-Israel Institute of Technology, Haifa, Israel; ^3^ Department of Hematology, VU University Medical Center, Amsterdam, The Netherlands; ^4^ Department of Clinical Genetics, VU University Medical Center, Amsterdam, The Netherlands; ^5^ Department of Pathology, VU University Medical Center, Amsterdam, The Netherlands; ^6^ Department of Experimental Therapeutics, Cancer Science Institute of Singapore, National University of Singapore, Singapore; ^7^ Texas Children's Cancer Center, Baylor College of Medicine, Houston, TX, USA; ^8^ Department of Epidemiology and Biostatistics, VU University Medical Center, Amsterdam, The Netherlands; ^9^ Department of Physiology, VU University, Amsterdam, The Netherlands; ^10^ Department of Rheumatology, Amsterdam Rheumatology and immunology Center, VU University Medical Center, Amsterdam, The Netherlands; ^11^ Current address: BGI-Shenzhen, Shenzhen, China

**Keywords:** bortezomib, resistance, proteasome, MARCKS, leukemia

## Abstract

PSMB5 mutations and upregulation of the β5 subunit of the proteasome represent key determinants of acquired resistance to the proteasome inhibitor bortezomib (BTZ) in leukemic cells *in vitro*. We here undertook a multi-modality (DNA, mRNA, miRNA) array-based analysis of human CCRF-CEM leukemia cells and BTZ-resistant subclones to determine whether or not complementary mechanisms contribute to BTZ resistance. These studies revealed signatures of markedly reduced expression of proteolytic stress related genes in drug resistant cells over a broad range of BTZ concentrations along with a high upregulation of myristoylated alanine-rich C-kinase substrate (MARCKS) gene expression. MARCKS upregulation was confirmed on protein level and also observed in other BTZ-resistant tumor cell lines as well as in leukemia cells with acquired resistance to other proteasome inhibitors. Moreover, when MARCKS protein expression was demonstrated in specimens derived from therapy-refractory pediatric leukemia patients (*n* = 44), higher MARCKS protein expression trended (*p* = 0.073) towards a dismal response to BTZ-containing chemotherapy. Mechanistically, we show a BTZ concentration-dependent association of MARCKS protein levels with the emergence of ubiquitin-containing vesicles in BTZ-resistant CEM cells. These vesicles were found to be extruded and taken up in co-cultures with proteasome-proficient acceptor cells. Consistent with these observations, MARCKS protein associated with ubiquitin-containing vesicles was also more prominent in clinical leukemic specimen with *ex vivo* BTZ resistance compared to BTZ-sensitive leukemia cells. Collectively, we propose a role for MARCKS in a novel mechanism of BTZ resistance via exocytosis of ubiquitinated proteins in BTZ-resistant cells leading to quenching of proteolytic stress.

## INTRODUCTION

The proteasome inhibitor Bortezomib (BTZ, Velcade^®^) is registered for the treatment of multiple myeloma (MM) and mantle cell lymphoma [1, 2] and is currently undergoing clinical evaluation in other hematological malignancies, such as pediatric acute leukemia [3]. Through reversible inhibition of the chymotrypsin-like activity of the β5 subunit and to a lesser extent the caspase-like activity of the β1 subunit of the 20S proteasome, BTZ specifically blocks proteasomal degradation of ubiquitinated proteins [4, 5]. Consequently, misfolded and poly-ubiquitinated proteins accumulate thereby activating the unfolded protein response (UPR) [6, 7]. The balance between protein production and the extent of degradation inhibition plays a key role in the cytotoxic activity exerted by BTZ [7]. Moreover, inhibition of multiple pro-survival pathways [8–13] contribute to the BTZ induced apoptosis. Despite the proven efficacy of BTZ, development of drug resistance is an emerging obstacle [14]. Although the mechanism underlying BTZ resistance in hematological malignancies is only partly understood, several proteasomal and non proteasomal-related factors involved in BTZ resistance have been suggested [14–17]. With respect to the latter, upregulation of heat shock proteins [18–20], drug extrusion by P-glycoprotein [21–23], upregulation of P21(WAF1/CIP1) [24], activation of the AKT/mTOR pro-survival pathway [25], downregulation of XBP1s [26], decreased CIP2A activity [27] and Noxa/Bcl-2 protein interactions [28] have been suggested to be implicated in BTZ-resistance. Proteasome-related mechanisms of BTZ resistance are associated with differential upregulation of β5 subunit along with downregulation of immunoproteasome subunit [21, 29–34]. Specifically, in acute leukemia cell lines and childhood ALL and AML leukemic blast cells, a lower ratio of immunoproteasome subunits over constitutive subunits was associated with a decreased sensitivity to BTZ [35]. Remarkably, reversing this balance towards increased immunoproteasome subunits upon treatment with interferon-γ exposure restored BTZ sensitivity in BTZ-resistant leukemic cells [31]. Moreover, acquisition of PSMB5 mutations and upregulation of mutated β5 subunits were frequently identified in hematological tumor and various solid tumor cell lines which conferred high levels of BTZ resistance *in vitro* [14, 17, 21, 29, 32, 36–39]. The identified mutations in PSMB5 form a cluster in a region that encodes for critical amino acids within or in close proximity to the BTZ- binding pocket of the β5 subunit resulting in decreased BTZ binding [29, 40]. Next generation proteasome inhibitors displayed differential capacities to overcome BTZ in hematological cells, but appeared themselves prone to the development of drug resistance by mechanisms including PSMB5 mutations [41, 42]. A currently open question is how BTZ-resistant cells harboring PSMB5 mutations handle proteolytic stress upon exposure of increasing BTZ concentrations. Examining the ability of BTZ to inhibit the catalytic activity of the mutated β5 subunit revealed a 2-fold lower potency as compared to non-mutated β5 subunits, whereas the cell growth inhibitory capacity was repressed by a factor of > 100 fold [29, 41]. These findings suggest that BTZ resistant cells acquired additional compensatory mechanism(s) to cope with the proteolytic stress. To gain further insight into these underlying molecular mechanisms, we undertook a multi-modality (DNA, mRNA, miRNA) array-based analysis of human CCRF-CEM leukemia cells and two subclones harboring PSMB5 mutations, one with a moderate and one with a high level BTZ resistance. These studies revealed a highly upregulated myristoylated alanine-rich C-kinase substrate (MARCKS) gene expression which correlated with protein expression. Moreover, MARCKS protein expression was associated with a BTZ concentration-dependent vesicular secretion of ubiquitinated proteins. The relevance of this novel function of MARCKs in BTZ resistance was further corroborated in BTZ and second generation proteasome inhibitor resistant hematological cell lines, *ex vivo* BTZ-resistant pediatric ALL cells, and clinical specimens of ALL children receiving BTZ-containing chemotherapy.

## RESULTS

To identify novel mechanisms of BTZ resistance, the human CCRF-CEM leukemia cell line and its BTZ-resistant sublines, i.e. CEM/BTZ7 (10-fold resistance), CEM/BTZ100 (140-fold resistance) and CEM/BTZ200 cells (170-fold resistance) [31, 43] were studied and analyzed in a multi-modality array-based analyses including comparative genomic hybridization (CGH), micro-RNA (miRNA) and gene expression (GEP) arrays.

### ArrayCGH analysis

ArrayCGH analyses of two BTZ-resistant subclones were compared to parental CEM/WT cells. Genetic alterations identified in CEM/BTZ7 cells included: a deletion of small area of the long arm of chromosome 5, a duplication of a large area on the end of the long arm of chromosome 11, a near complete duplication of the long arm of chromosome 14 as well as a complete loss of one of the three X-chromosomes (Supplementary Figure S1A). Of note, chromosome 14 harbors multiple proteasomal subunits, including *PSMB5* (β5) and *PSMA3* (α7) which we were previously shown to be upregulated at the protein level in the BTZ-resistant CEM lines [29]. In addition, a limited number of small duplications and deletions on different chromosomes were observed. Similar genetic alterations were identified in CEM/BTZ200 cells (Supplementary Figure S1B). Karyotype analysis of CEM/WT and CEM/BTZ200 cells confirmed the loss of chromosome X and duplication of chromosome 14 (Supplementary Figure S1C and S1D).

### miRNA array analysis

**Figure 1:**
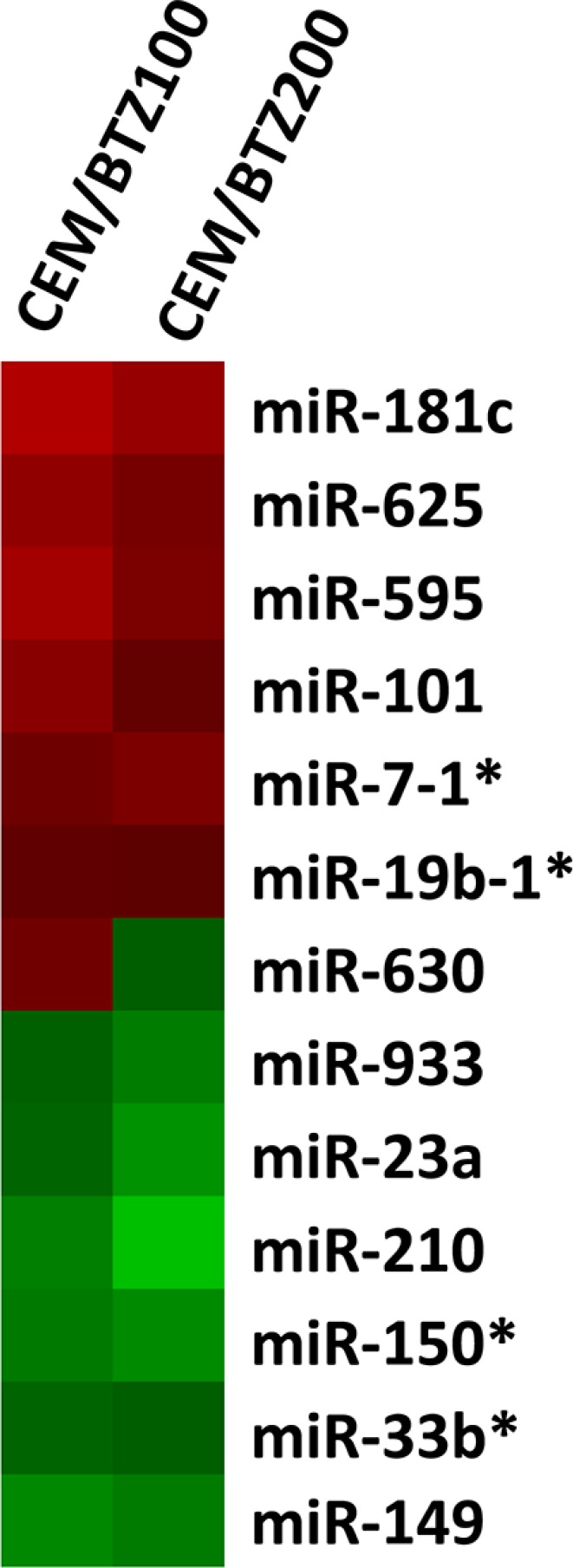
Differential miRNA expression between BTZ-resistant CEM cells and CEM/WT Red color represents upregulation, green color downregulation.

miRNA array analysis was performed to identify possible regulatory miRNAs involved in BTZ resistance. Figure 1 shows all differentially expressed miRNAs in CEM/BTZ100 and CEM/BTZ200 cells as compared to parental CEM/WT cells. Among the most down-regulated miRNAs were the hypoxia-induced miR-210 [43], the Myc down-regulated miR-23a [44], the hematological differentiation inducing miR-150 (reviewed in [45]) and the possible tumor suppressor miR-149 [46]. Of the upregulated miRNAs, miR-181c has been associated with cell proliferation [47, 48] and miR-19b has been correlated with 5-FU resistance [49]. In contrast to these miRNAs supporting pro-survival, two other upregulated miRNA's have been described to have the opposite effect. miR- 101 has been described to be a pro-apoptotic factor in childhood acute lymphoblastic leukemia [50] and miR-7 as an tumor suppressor inhibiting various receptor tyrosine kinases such as EGFR [51], IGF-1R [52] and p21 activated kinase (PAK1) [53]. miR-29b, which was recently shown to target the proteasome subunit PSME4 and disrupt the autophagosome pathway in BTZ-resistant MM cells [54], was not down-regulated in CEM/BTZ cells, indicating non-overlapping profiles in BTZ-resistant acute leukemia and MM cells. An overview of expression validated target genes of the differentially expressed miRNAs is presented in Supplementary Table S1. Differentially expressed miRNAs were not located on amplified or deleted genomic regions identified in the arrayCGH analysis.

### Gene expression profile (GEP) analysis

**Figure 2:**
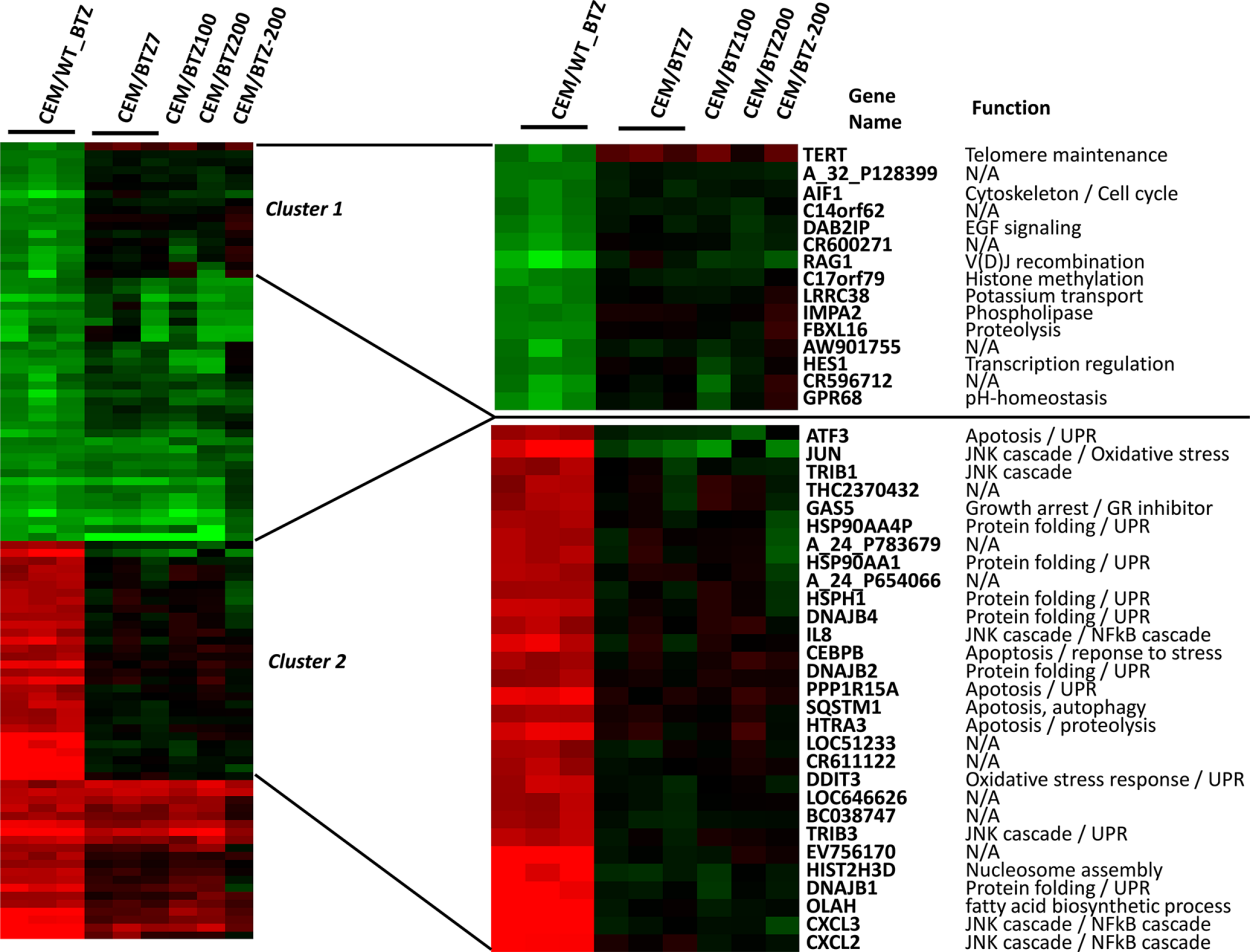
Top 50 upregulated and top 50 significantly down-regulated genes (*p* < 0.05) comparing CEM/WT_BTZ (CEM/WT cells incubated for 24 hrs with 7 nM BTZ) to CEM/BTZ7 (CEM cells resistant to 7 nM BTZ) The figure depicts this selection of genes for all CEM variations as a ratio of the untreated CEM/WT cells. (Clustering performed using Cluster 3.0) An overview of top 50 upregulated and top 50 down-regulated genes is depicted. For genes in cluster 1 and cluster 2, gene annotation and function are provided.

In order to identify differences in response to BTZ exposure in sensitive and BTZ resistant CEM cells, parental CEM/WT cells were treated with 7 nM BTZ for 24 hours (CEM/WT_BTZ) and compared to the different resistant sublines, treated for 24 hours with a concentration of BTZ on which they normally thrive (CEM/BTZ7 with 7 nM, CEM/BTZ100 with 100 nM and the CEM/BTZ200 with 200 nM of BTZ). Ratios of gene expression were calculated as compared to untreated parental CEM/WT. Figure 2 shows the 50 most upregulated and downregulated genes after treatment of CEM/WT cells with BTZ for 24 hours. After clustering, 2 groups with highly differentially expressed genes between CEM/WT_BTZ and all resistant subtypes were identified. The right top side of Figure 2 shows a closer view of cluster 1, containing genes highly down-regulated in CEM/WT_BTZ cells which were essentially unchanged in the BTZ-resistant CEM sublines. The functions of the genes in cluster 1 were diverse and affected pathways including: cell proliferation, immune response, TGF-β pathway and transcriptional regulation. When focusing on cluster 2, the genes upregulated in the CEM/WT_BTZ and unchanged in the BTZ-resistant cell lines are nearly all involved in UPR, apoptosis or JNK cascade signaling (Figure 2 right, bottom). Clearly, the stress response observed in the CEM/WT cells by BTZ was not observed in the BTZ-resistant CEM cells.

**Figure 3:**
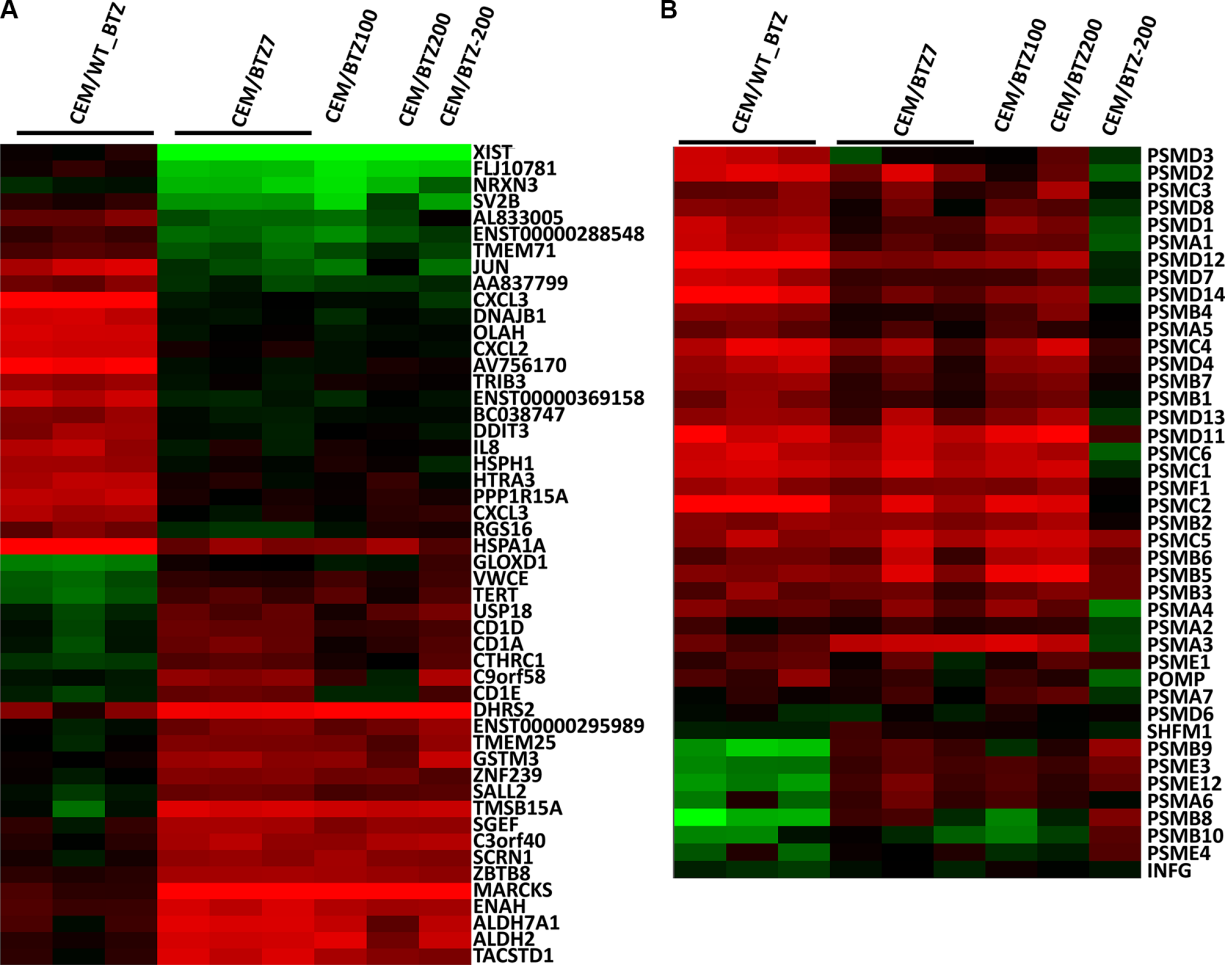
(**A**) Top 20 up- and downregulated genes (*p* < 0.05) and (**B**) proteasomal genes comparing CEM/WT_BTZ with CEM/BTZ7 (*p* < 0.05). Results for all CEM variations are depicted as a ratio of the untreated CEM/WT. Red color represents upregulation, whereas green downregulation of expression.

Of the highest differentially expressed genes comparing the CEM/WT_BTZ to the CEM/BTZ7, several genes overlapped with the genes found in clusters 1 and 2 (Figure 2) and several other genes were identified (Figure 3A). Among the genes that were down-regulated in the BTZ-resistant CEM lines as compared to the CEM/WT_BTZ cells were several stress-related genes (including DNAJB1, DDIT3, HSP1 and HSPA1A) and immune-related genes (including CXCL2, CXCL3, and IL-8). The most down-regulated gene was XIST, which resides on the X-chromosome and which is in concordance with our array CGH analysis.

The list of upregulated genes contains several genes encoding for enzymes involved in detoxification (including ALDH7A1, ALH2, GSTM3 and DHRS2), CD1 genes (CD1A, CD1D and CD1E), the cell cycle gene TERT and several genes involved in cytoskeleton regulation and vesicle migration (ENAH, SCRN1 (SES1), SGEF, TMSB15A, C9orf58 (AIF1L) and MARCKS. Of all genes, MARCKS (myristoylated alanine-rich C-kinase substrate) was the most differentially overexpressed gene with a 25- to 42-fold upregulation in the BTZ-resistant leukemia cell lines.

It has been shown that BTZ induces upregulation of multiple proteasomal genes after short-term drug exposure [10]. The expression of constitutive proteasomal genes in CEM/WT cells was upregulated upon BTZ exposure (Figure 3B). Interestingly, only a small difference was observed in proteasomal gene expression when comparing parental cells and BTZ-resistant tumor cell lines after 24 hours of BTZ treatment. Upon BTZ treatment, CEM/WT cells displayed an upregulation of the constitutive proteasome along with down-regulation of the immune proteasome (i.e. subunits PSMB8, PSMB9 and PSMB10). In BTZ-resistant cell lines, apart from upregulation of the constitutive proteasome, expression of immune proteasome was largely normalized to CEM/WT levels.

To gain more insight into the interaction between upregulated and downregulated genes, pathway as well as gene set enrichment analysis was performed. Using the GeneGo tool, several protein stress pathways known to be involved in BTZ-induced cytotoxicity [6, 7, 55], including “apoptosis and survival endoplasmic reticulum stress response” and “protein folding response to proteins stress” were identified showing upregulation of several genes in CEM/WT_BTZ as compared to untreated parental CEM/WT cells (Supplementary Figure S2A and S2B). When CEM/BTZ7 was compared with CEM/WT_BTZ, a downregulation of these pathways was observed (Supplementary Figure S2A and S2C). An overview of the top identified affected network processes including these protein stress pathways when comparing CEM/WT_BTZ to the untreated parental CEM/WT cells is shown in Table 1 (left column). In contrast, when the resistant CEM/BTZ7 cells were compared with CEM/WT_BTZ, the opposite trend was observed in these pathways (Table 1, right column) indicating a lack of stress in these BTZ-resistant cells.

**Table 1:** Overview of network processes identified using GeneGO pathway analysis based on GEP

CEM/WT_BTZ vs CEM/WT Process –networks	*P* value	CEM/BTZ7 vs CEM/WT_BTZ Process –networks	*P* value
Protein folding_Response to unfolded proteins	9,87E-11	Protein folding_Response to unfolded proteins	6,19E-11
Protein folding_Folding in normal condition	4,06E-07	Apoptosis_Endoplasmic reticulum stress pathway	1,17E-05
Apoptosis_Endoplasmic reticulum stress pathway	3,34E-05	Protein folding_Folding in normal condition	2,42E-05
Apoptosis_Apoptotic mitochondria	1,27E-03	Apoptosis_Apoptotic mitochondria	2,60E-04
Protein folding_Protein folding nucleus	3,20E-03	Reproduction_Male sex differentiation	7,11E-04
Proteolysis_Ubiquitin-proteasomal proteolysis	8,27E-03	Immune response_Th17-derived cytokines	1,12E-03
Inflammation_IL-6 signaling	8,34E-03	Reproduction_FSH-beta signaling pathway	1,21E-03
Inflammation_Neutrophil activation	9,02E-03	Protein folding_Protein folding nucleus	2,58E-03
Cytoskeleton_Intermediate filaments	1,05E-02	Inflammation_IL-6 signaling	3,43E-03
Protein folding_ER and cytoplasm	1,18E-02	Reproduction_Spermatogenesis, motility and copulation	4,45E-03

Corresponding *P*-values are given for the identified pathways. Pathways analysis was performed using GeneGO on significant altered genes (*P* < 0.05) and with a fold change > Log2.

Gene Set Enrichment Analysis (GSEA) confirmed upregulation of several stress-related gene sets in CEM/BTZ cells, including the set of genes most significantly changed after exposure to the proteasome inhibitor epoxomicin (gene set CONCANNON_APOPTOSIS_BY_EPOXOMICIN_UP [56]). Proliferation, protein metabolism and MYC-related gene sets were down-regulated. In contrast, the CEM/BTZ7 gene expression profile was enriched for MYC-regulated genes and did not show upregulation of protein stress gene sets. (Supplementary Table S2). In addition, miRNA expression was correlated with GEP. In this selected gene list, pathway analysis was performed as well. An overview of the highly affected pathways is depicted in Table 2. Apart from comparable pathways including protein handling and apoptosis, several cytoskeleton-associated pathways were identified. Collectively, these data indicate that ER stress is most likely to be the dominant mechanism of BTZ-induced cytotoxicity in parental CCRF-CEM cells. Whereas, the diminished stress observed in drug resistant CEM/BTZ7 cells suggests a novel resistance mechanism circumventing the UPR.

**Table 2:** Overview of process-networks and gene ontology processes based on miRNA analysis

Process -networks	*P*-value	GO-Processes	*P*-value
Proteolysis_Ubiquitin-proteasomal proteolysis	2,95^E^-03	Apoptotic process	1,35^E^-07
Protein folding_Protein folding nucleus	6,19^E^-03	Programmed cell death	2,23^E^-07
Cell adhesion_Leucocyte chemotaxis	1,51^E^-02	Cellular response to stress	2,63^E^-07
Cytoskeleton_Regulation of cytoskeleton rearrangement	1,64^E^-02	Regulation of molecular function	1,21^E^-06
Cell adhesion_Synaptic contact	1,71^E^-02	Cell death	1,48^E^-06
Cell adhesion_Cell junctions	1,84^E^-02	Cellular response to oxidative stress	1,55^E^-06
Cytoskeleton_Intermediate filaments	2,89^E^-02	Death	1,68^E^-06
Cytoskeleton_Actin filaments	3,06^E^-02	Cellular protein metabolic process	1,73^E^-06
Apoptosis_Apoptotic nucleus	3,99^E^-02	Cellular protein modification process	2,47^E^-06
Reproduction_Progesterone signaling	4,36^E^-02	Protein modification process	2,47^E^-06

Corresponding *P*-values are given for the identified pathways. Pathways analysis was performed using GeneGO on targets genes of the top10 miRNA's of which expression is correlating with gene expression.

### MARCKS upregulation

**Figure 4:**
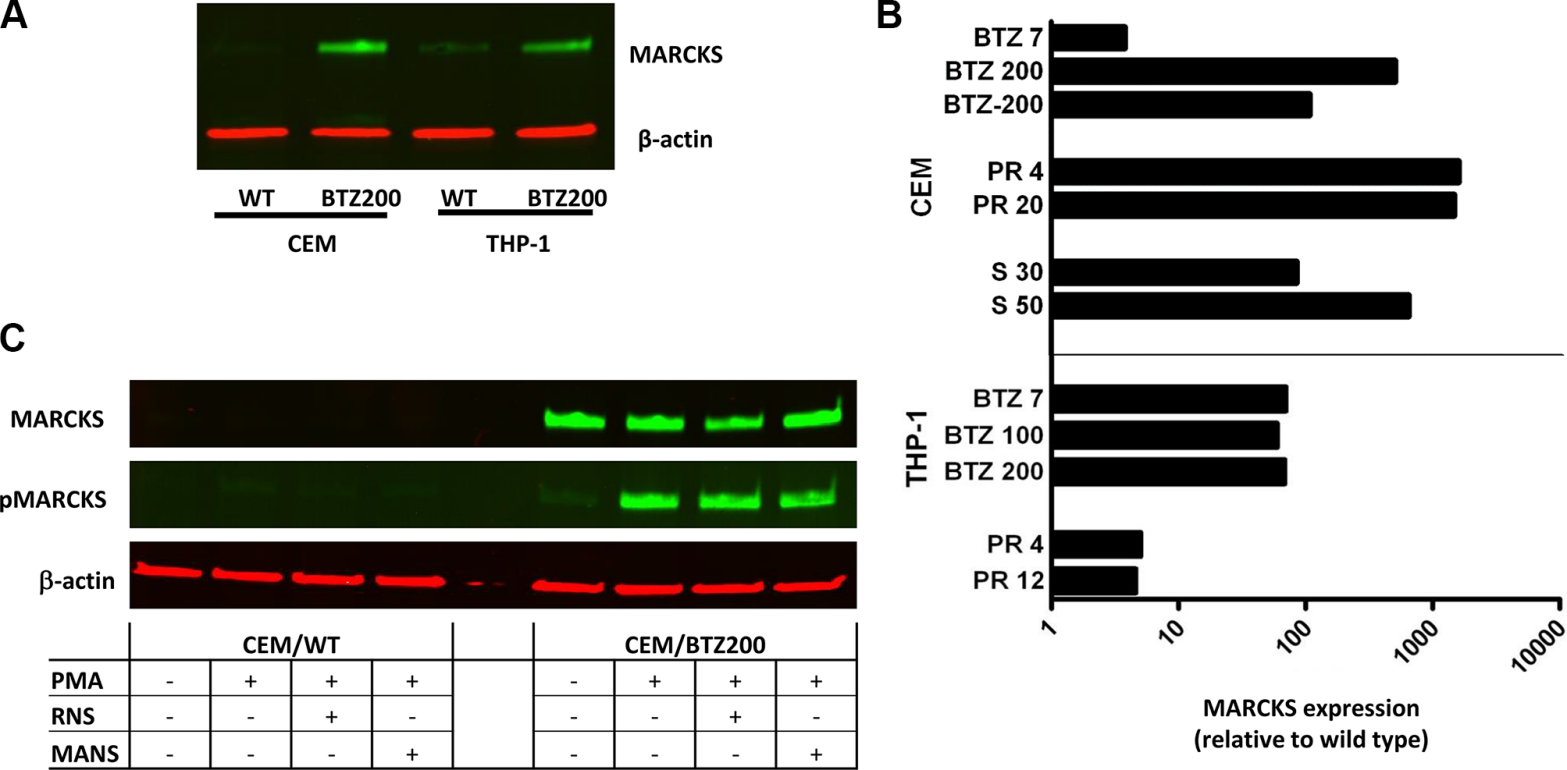
(**A**) MARCKS protein expression of CEM and THP-1 cells and their BTZ resistant sublines CEM/BTZ200 and THP-1/BTZ200. In addition, the CEM/BTZ200 cultured in absence of BTZ for several months (CEM/BTZ-200) is also included. Western blot analysis of MARCKS (green) and β-actin (red) as a loading control, **(B)** Ratio of the quantification of MARCKS protein expression (β-actin normalized) of proteasome inhibitor (PR924 and Marizomib) resistant THP-1 and CEM cells relative to their parental cells. **(C)** Basal levels of phosphorylated MARCKS expression in CEM/WT and CEM/BTZ cells before and after incubation with PMA (50 nM for 1 hour,), and a combination of PMA and RNS (1 hour 100 nM) or MANS (1 hour 100 μM).

To confirm the upregulation of MARCKS in BTZ-resistant leukemia cell lines at the protein level, Western blot analysis was performed as described previously [29]. Figure 4A shows a marked 500 fold upregulation of MARCKS in CEM/BTZ200 cells as compared to parental CEM cells. Consistently, a 60 fold upregulation was found in the previously described BTZ-resistant AML cells (THP-1/BTZ200) [32] as compared to THP-1/WT (Figure 4A). Remarkably, MARCKS was also upregulated in the recently established CEM and THP-1 (1500 fold and 5 fold respectively) sublines with acquired resistance to the immunoproteasome inhibitor PR-924 [42] and a CEM subline (630 fold) with acquired resistance to the proteasome inhibitor salinosporamide A (marizomib) [41] (Figure 4B). Next to MARCKS expression, levels of phosphorylated MARCKS were examined in CEM/WT and CEM/BT200 cells. Notably, CEM/BTZ200 cells expressed a low basal level of phosphorylated MARCKS, which was inducible upon stimulation of cells with PMA and only partially blocked (35% pMARCKS reduction) by treatment with MANS [57] (Figure 4C).

### Vesicle-mediated ubiquitin exocytosis

**Figure 5:**
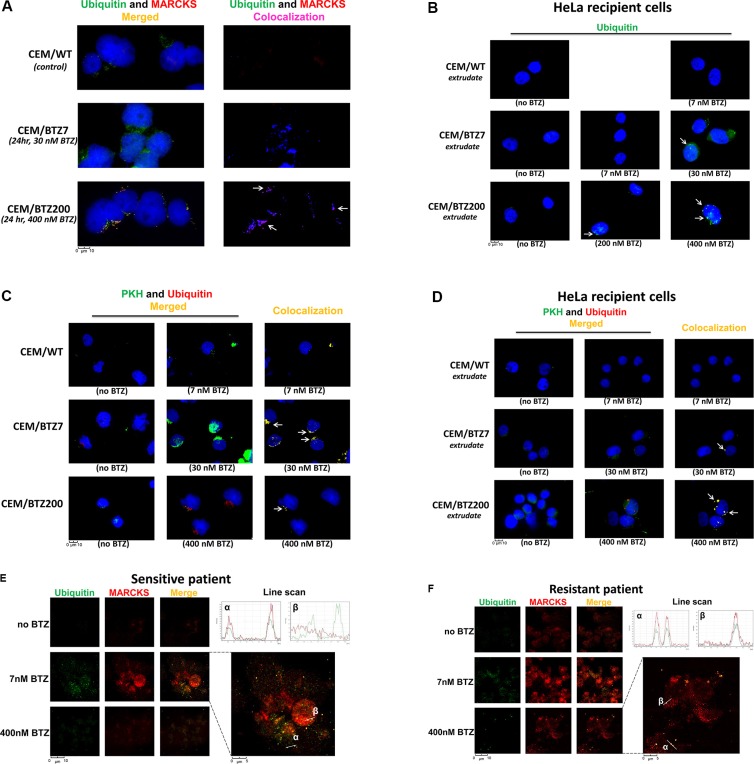
Fluorescence microscopy of vesicles staining in WT and BTZ-resistant CEM cells, primary patient samples, and HeLa cells (**A**) MARCKS and ubiquitin protein expression in CEM/WT cells, CEM/BTZ7 cells treated with 30 nM BTZ for 24 hours and CEM/BTZ200 cells treated with 400 nM BTZ for 24 hours. Left panel: DAPI nuclear staining (Blue), ubiquitin (Green), and MARCKS (Red), right panel: level of co-localization of MARCKS with ubiquitin as calculated by the SlideBook microscope software as depicted in blue (normal) to magenta (strong co-localization). Single channel images are shown in Supplementary Figure S4. (**B**) Ubiquitin (green) and DAPI (blue) staining in HeLa cells after 24 hour incubation with the supernatants of cultures of CEM/WT, CEM/BTZ7 and CEM/BTZ200 cells untreated or treated with the indicated concentrations of BTZ for 1 hour. (**C**) PKH labeling and ubiquitin protein expression in CEM/WT cells, CEM/BTZ7 and CEM/BTZ200 cells treated with the abovementioned concentrations of BTZ for 24 hours. DAPI nuclear staining (Blue), PKH (Green), and ubiquitin (Red) and level of colocalization of MARCKS with ubiquitin as calculated by the SlideBook microscope software as depicted in blue to magenta (Right panel). (**D**) Ubiquitin (red) and PKH (green) staining in HeLa cells after 24 hour incubation with the supernatants of cultures of CEM/WT, CEM/BTZ7 and CEM/BTZ200 cells untreated or treated with the indicated concentrations of BTZ. Calculated co-localization is depicted in yellow. (**E**) Ubiquitin (green) and MARCKS (red) staining in a primary ALL patient samples, one sensitive to BTZ (LC50: BTZ 6 nM) and one resistant to BTZ (LC50 BTZ: 262 nM) (**F**) without and after incubation with low concentration (7 nM) or high concentration of BTZ (400 nM). In the merge figure, the co-localization of the two proteins is shown in yellow. The right top of the figures depict a quantification using a line scan through the corresponding area of interest (α and β).

MARCKS protein was previously shown to be involved in multiple exocytosis pathways [58], e.g. mucin secretion [59], mast cell degranulation [60] and membrane targeting of plasmalemmal precursor vesicles during axon development [61]. Since gene expression profiling studies for CEM/BTZ cells (Figure 3) revealed that apart from MARCKS, several other genes involved in exocytosis, mostly cytoskeleton rearrangement genes, were also upregulated, we hypothesized that vesicle-mediated export of accumulated ubiquitinated proteins contributes to BTZ resistance through bypassing the proteasome and the UPR. To explore this hypothesis we first investigated whether or not the ubiquitinated proteins co-localized with MARCKS in vesicles in BTZ-resistant sublines. We subsequently determined the secretion of vesicles by BTZ-resistant sublines by following the uptake of vesicles by recipient HeLa cells after 24 hour exposure to supernatant derived from BTZ-resistant sublines. Figure 5A depicts the results of the background staining in the untreated parental CEM/WT showing hardly any MARCKS protein and moderate levels of ubiquitin. After 24 hour exposure of CEM/BTZ7 cells to 30 nM BTZ, MARCKS was upregulated when compared to parental CEM/WT cells. Moreover, ubiquitin was increased, partly in vesicular structures, and partly as a diffuse cytoplasmic distribution. CEM/BTZ200 cells exposed to 400 nM BTZ displayed a clear co-localization of ubiquitin with MARCKS mostly in vesicle-like structures (Figure 5A and Supplementary Figure S4). Of note, co-localization was not observed with phosphorylated MARCKS (not shown). After exposure of HeLa cells to supernatant of CEM/WT cells we performed immunocytochemical staining to detect possible ubiquitin-containing vesicles (Figure 5B). These vesicles were not identified in HeLa cells exposed to supernatants of any of the untreated CEM/WT or BTZ-resistant sublines. When CEM/BTZ7 cells were exposed to 7 nM BTZ, a drug concentration on which they normally thrive, no uptake of vesicles was noted in HeLa cells. However, when CEM/BTZ7 cells were more stringently stressed with 30 nM of BTZ, uptake of ubiquitin-containing vesicles started to appear; the most pronounced uptake of ubiquitin-containing vesicles by HeLa cells was observed after the addition of a supernatant of CEM/BTZ200 cells treated with 400 nM BTZ. Further support to the hypothesis that the secretion of ubiquitin is indeed vesicle-mediated, was achieved by PKH67 staining. Figure 5C shows co-localization of ubiquitin and PKH in CEM/BTZ7 and CEM/BTZ200 after treatment with 30 nM and 400 nM BTZ, respectively. Moreover, when supernatants of these cell cultures were added to HeLa cells, co-localization of ubiquitin and PKH was observed in recipient cells (Figure 5D), indicating the uptake of ubiquitin-containing vesicles. No uptake was seen in untreated WT and BTZ-resistant cells. Taken together, these data strongly suggest that the ER stress that is normally observed after aggresome formation of aggregated ubiquitinated proteins is circumvented in BTZ-resistant CEM cells by exocytosis of vesicles containing the aggregated ubiquitinated proteins.

To confirm these findings for primary patient samples, we selected a pediatric ALL patient displaying *ex vivo* sensitivity to BTZ (LC50: 6 nM, Figure 5) and compared it to a BTZ-resistant patient sample (LC50: 262 nM). Figure 5E and 5F show MARCKS and ubiquitin expression in these leukemic cells after 24 h exposure to a low dose (7 nM) and a high dose (400 nM) of BTZ. At low BTZ concentrations, the sensitive patient sample readily shows high ubiquitin and MARCKS expression with some co-localization. When this sample was incubated with high concentration of BTZ, vitality of the cells was heavily compromised along with diffuse staining of both proteins. The BTZ-resistant patient sample exposed to a low dose of BTZ shows some diffuse upregulation of ubiquitin as well as MARCKS expression. When this sample was incubated with 400 nM BTZ, ubiquitin and MARCKS show clear co-localization in small vesicular structures inside as well as outside the cell. The level of co-localization is quantified in the corresponding line scan. Together, these data show that the phenomenon of ubiquitin exocytosis by BTZ-exposed cells is also observed in primary patient samples.

### MARCKS and BTZ resistance

We assessed whether PMA-stimulation of MARCKS phosphorylation or inhibition of MARCKS phosphorylation by either the inhibitory peptide MANS [62] or inhibition of protein kinase C (PKC) activity by staurosporine or UCN01 had an impact on BTZ sensitivity in CEM/BTZ200 cells. However, neither of these conditions appeared to alter BTZ sensitivity (Supplementary Figure S3; (A); BTZ dose response curve after PMA stimulation or MANS inhibition; (B) co-incubation with a concentration range of UCN-01 or (C) staurosporine). Moreover, siRNA knockdown of MARCKS by 56% (Supplementary Figure S3D) did not influence BTZ sensitivity (Supplementary Figure S3E; BTZ dose response curve after MARCKS siRNA). These data do not support a causal role of MARCKS in the described exocytosis mechanism and show that partial knockdown of MARCKS is insufficient to restore BTZ sensitivity.

### Clinical prognostic value of MARCKS expression

**Figure 6:**
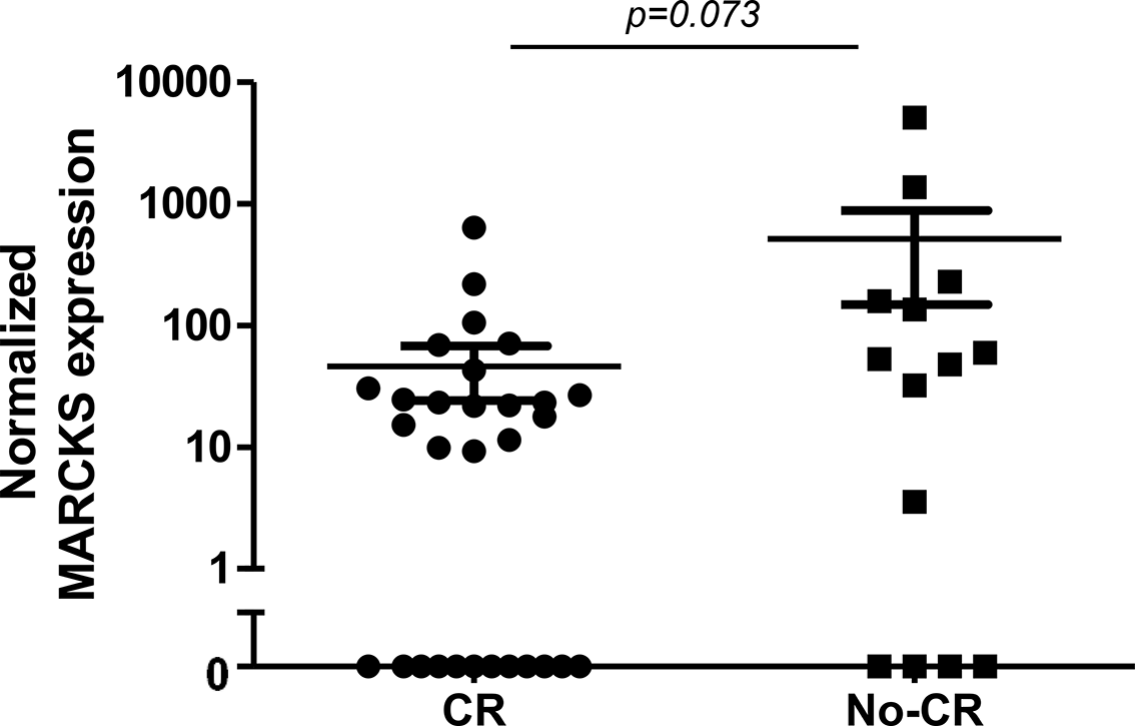
Pre-treatment MARCKS protein expression (Western blot) in primary ALL samples stratified according to response to BTZ-containing treatment (Complete remission (CR) after induction chemotherapy vs no CR) Data are presented after normalization to β-actin. For both groups, the mean and standard error of the mean (SEM) is depicted.

Given the overexpression of MARCKS in BTZ-resistant leukemia cells, we explored whether or not MARCKS overexpression may serve as predictive marker of BTZ unresponsiveness in clinical samples of acute leukemia patients. To this end, we examined primary leukemic specimens of patients from the phase II childhood refractory/relapsed ALL trial (AALL07P1 study, NCT00873093) in which BTZ is administered in two intensive re-induction regimens containing vincristine, prednisone, PEG-asparaginase, doxorubicin or cyclophosphamide and etoposide followed by methotrexate treatment. In these pretreatment samples we determined MARCKS expression levels using Western blot analysis and explored the correlation with the clinical response [Complete remission (CR) or no complete remission (No-CR)]. Figure 6 shows MACRKS expression in the CR and no-CR groups (β-actin and CEM/WT normalized). Twelve out of 30 patient specimens (40%) in the CR group did not show MARCKS expression when compared to 4 out of 14 (29%) in the no-CR group. MARCKS expression in the CR group was lower as compared with no-CR group (median 13.4 vs 50.3), with a trend for statistical significance (*P* = 0.073).

## DISCUSSION

The current study reports a novel mechanism of exocytosis-mediated extrusion of vesicle-like structures containing ubiquitinated proteins in BTZ-resistant CEM leukemia cells as a novel modality to overcome proteolytic stress over a broad range of cytotoxic BTZ concentrations. MARCKS protein was identified as a BTZ resistance biomarker associated with the intracellular emergence of these vesicle-like structures. Furthermore, ubiquitin-containing vesicles from supernatants of BTZ-treated resistant CEM/BTZ cells were taken up by HeLa cells, hence confirming actual exocytosis of the ubiquitin-containing vesicles. Thus, through exocytosis of ubiquitinated proteins, the proteasomal load is decreased during proteasome inhibition, leading to cell survival. As proof of principle, this phenomenon was confirmed in two primary ALL patient samples. To our knowledge, this is the first report showing an association between resistance to proteasome inhibitors and exocytosis of ubiquitinated proteins.

The concept of disposal of abundant ubiquitinated proteins is supported by our gene expression profiling and miRNA data. It is well established that accumulation of polyubiquitinated proteins as a result of proteasome inhibition induces an unfolded protein response (UPR) in several tumor models [6, 14, 63–65]. Pathway analysis of BTZ-treated CEM/WT cells also showed enrichment for unfolded protein response and ER-stress pathways. Interestingly, upon BTZ exposure, BTZ-resistant leukemia cell lines showed a major reduction of active protein stress-related pathways, indicating a resistance mechanism circumventing UPR. This phenomenon has also been found to be reduced in BTZ-resistant mantle cell lymphoma (MCL) [66] and multiple myeloma [67, 68]. Apart from stress-related pathways, correlation of miRNA expression with gene expression identified cytoskeleton-associated pathways as being altered in BTZ-resistant CEM cells (Table 2). This latter pathway is involved in transport of vesicles, hence lending further support to the exocytosis hypothesis (reviewed in [69]). In addition, pan-genomic profiling of CEM/BTZ cells confirmed the genetic basis of the previously reported upregulated expression of mutated PSMB5 (β5) as a mechanism of BTZ resistance [29].

Apart from proteasomal degradation, the endosomal sorting complexes required for transport (ESCRT) pathway and autophagy have been described in transport and selective removal of ubiquitinated proteins. In the ESCRT pathway, membrane proteins are taken up in endosomes and fuse with lysosomes resulting in the degradation of its content [70]. However, since ubiquitin is removed from membrane proteins before endocytosis through the ESCRT, ubiquitin-containing vesicles would not be present. Hence, this mechanism is not likely to be involved in the BTZ-resistant CEM cells.

Secondly, under conditions of proteasome protein overload or energy deprivation, proteins and intact organelles can be degraded by lysosomes through autophagy [71]. In the context of proteasome inhibition or overload, autophagy of ubiquitinated protein aggregates is of interest [72, 73]. While autophagosomes normally fuse with lysosomes resulting in degradation of the aggresome, exocytosis of phagosomes has been opted in a process called exophagy [74]. Although increased autophagy, mediated by increased HSPB8 expression, has recently been demonstrated to be involved in BTZ resistance in multiple myeloma cells [20], we found no increased activity of this pathway in BTZ-resistant CEM cells. Our GEP data showed no upregulation of autophagy-related proteins, including HSPB8, while even a downregulation of the autophagy initiator SQSTM1 was seen (data not shown). In addition, the autophagy marker LC3B was not increased in BTZ-resistant cell lines as determined by Western blot and immunocytochemistry (data not shown) and hence does not explain the phenomenon of vesicular exocytosis of ubiquitinated proteins we describe in this paper.

Buschow *et al.* [75] recently showed exosomes that were relatively enriched for ubiquitinated proteins as compared to total cell lysates [76]. Although their model was postulated, the exact mechanism of biogenesis and release of these exosomes was not elucidated. Since autophagy/exophagy and an ESCRT-dependent pathway are not supported by our data, BTZ- resistant CEM cells may have adopted the activation of an ESCRT-independent pathway as has been described by Buschow et al. By PKH labeling, ubiquitin-containing vesicles/exosomes emerging in CEM/BTZ cells during increasing BTZ concentrations were characterized by double membrane structures which remained intact during cellular release, interaction and accumulation in HeLa recipient cells. As such, these cells which are proteasome activity proficient have the capacity to process polyubiquitinated proteins.

Our GEP studies identified the MARCKS gene to be highly overexpressed in BTZ-resistant CEM cells. The association of MARCKS protein with BTZ resistance was originally identified by Micallef *et al.* [77] in MM RPMI 8226-R5 cells with acquired resistance to the farnesyltransferase inhibitor R115777 and 3-fold cross-resistant to BTZ [78]. Unlike CEM/BTZ cells, RPMI8226-R5 cells had no mutations in the *PSBM5* gene. Recently, Yang *et al.* [79] showed that upregulation of phosphorylated MARCKS in three BTZ-resistant multiple myeloma cell lines as well as in primary resistant MM specimens. They showed that low levels of BTZ resistance achieved through MARCKS regulating the SKP2/p27Kip1 cell cycle pathway [79, 80]. Consistently, in leukemia cells we show here the upregulation of MARCKS protein in CEM/BTZ cells as well as CEM cells with acquired resistance to new generation proteasome inhibitors Marizomib and PR924 [41, 42]. However, unlike MM cells, in leukemia cells we noted marginal basal levels of MARCKS phosphorylation suggesting no major involvement in leukemia cells that were selected for BTZ resistance. Notably, high levels of unphosphorylated MARCKS may be concordant with other well established functions of MARCKS in secretion processes [58–62]. Specifically, membrane-bound unphosphorylated MARCKS, rather than cytoplasmic phosphorylated MARCKS, can be a partner protein in facilitating transport of vesicles along the cytoskeletal axis and their secretion [61, 81]. This function would be consistent with co-localization studies of intracellular MARCKS and ubiquitin-containing vesicles and MARCKS not being associated with secreted vesicles. To this end, our encouraging preliminary correlations of MARCKS expression and BTZ response in pediatric acute leukemia patients warrant follow up study in a large cohort of BTZ-treated ALL.

**Figure 7:**
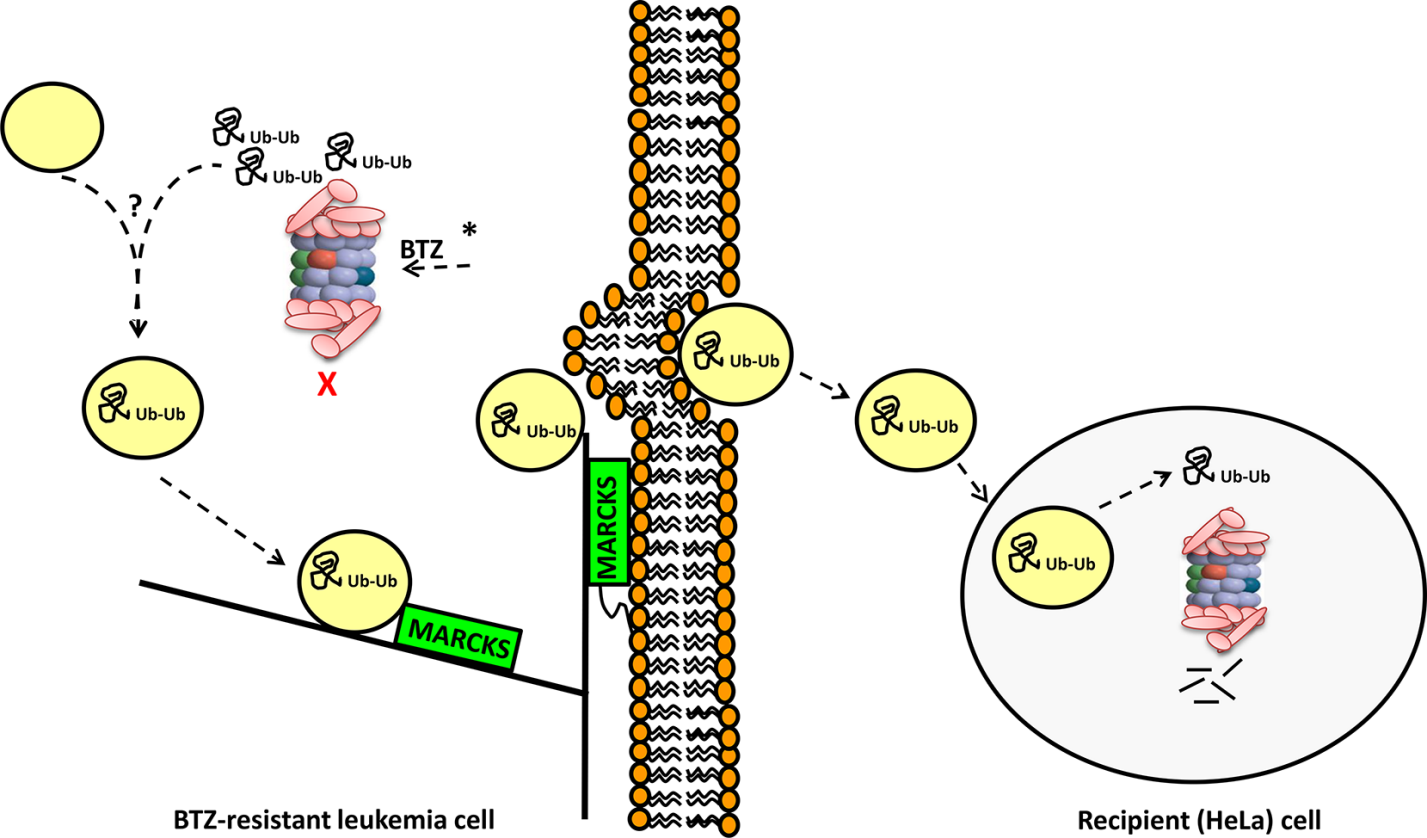
Summary model of the involvement of MARCKS and vesicular exocytosis of ubiquitinated proteins in BTZ-resistant leukemia cells CEM/BTZ cells harboring PSMB5 mutations (indicated by *) have a diminished capacity of inhibition of proteasomal catalytic activity by BTZ [29, 31]. Upon exposure of CEM/BTZ7 and CEM/BTZ200 cells to BTZ concentrations that block PSMB5 activity (30 nM and 400 nM BTZ, respectively), these cells accumulate polyubiquitinated proteins. This coincides with the biogenesis of vesicle-like structures incorporating these ubiquitinated proteins. These vesicles then traffic along actin/cytoskeleton axis to the plasma membrane with MARCKS protein serving as partner protein. Here myristoylated-anchored MARCKS facilitates exocytosis of vesicles which subsequently can be taken up by recipient (HeLa) cells. Proficient proteasomal activity in recipient cells would allow degradation of ubiquitinated proteins from BTZ-resistant cells. Through a mechanism of exocytosis-mediated extrusion of vesicles containing ubiquitinated proteins, BTZ-resistant cells can overcome proteolytic stress over a broad range of BTZ concentrations.

In conclusion, BTZ resistant T-ALL CEM cells displaying upregulation of mutant PSMB5, have a coexisting novel mechanism of exocytosis of ubiquitinated proteins, hence circumventing UPR (Figure 7). Both mechanisms are readily observed in resistance to several proteasome inhibitors indicating common resistance adaptation to proteasome inhibitors rather than limited to BTZ only. Further research is warranted to determine whether or not MARCKS is a clinical biomarker which can predict proteasome inhibitor resistance and may aid to select patients for BTZ- containing treatment strategies.

## MATERIALS AND METHODS

### Antibodies and chemicals

Anti-β-actin (clone c4) mouse mAb was purchased from Boehringer Mannheim (Almere, The Netherlands), α-Tubulin (B-7) mouse mAb and Ubiquitin (P4D1) mouse mAb from Santa Cruz Biotechnology (Santa Cruz, CA, USA), MARCKS (D88D11) XP^®^ Rabbit mAb #5607 and phospho-MARCKS (D13D2) (S159/S163) Rabbit mAb #11992S from Cell Signaling (Danvers, MA, USA), the IRDye infrared secondary labeled antibodies from LI-COR Biosciences (Lincoln, NE, USA) and the Polyclonal goat anti-mouse Immunoglobulins/FITC (Goat F(ab')2) from Dako (Glostrup, Denmark).

Trypsin was purchased from Lonza (Basel, Switzerland), PKH67 labeling kit, paraformaldehyde (PFA), Triton-X100, 4′, 6-diamidino-2-phenylindole (DAPI) and phorbol-myristic acid (PMA) from Sigma-Aldrich (Zwijndrecht, The Netherlands). BTZ was obtained via the VUmc hospital pharmacy department. MARCKS inhibitory peptide (MANS) was from Genemed Synthesis Inc (San Antonio, Texas, USA).

### Human tumor cell lines and patient samples

The generation, cell culture conditions and mutation status of the *PSMB5* gene of human leukemic CCRF-CEM cells and BTZ-resistant sublines, selected at 7, 100 and 200 nM BTZ, was previously described [29]. HeLa cells were obtained from ATCC (Manassas, VA, USA). Pre-treatment primary leukemic specimens were obtained from patients included in the phase II childhood refractory/relapsed ALL trial (AALL07P1 study, NCT00873093). After thawing the vials blast percentage was determined using May-Grunwald/Giemsa staining. Inclusion criteria for analysis was a blast percentage of > 20%. The samples were snap-frozen for MARCKS protein analysis.

### Karyotyping

Karyotyping was performed on GTG banded metaphase cells and described according ISCN 2013 [82].

### Microarray comparative genome hybridization (arrayCGH) for DNA copy number analysis

### Sample preparation and DNA isolation

Prior to DNA isolation, BTZ-resistant sublines were cultured in bortezomib-free medium for a week. DNA isolation was performed using column-based method (QIAamp DNA Mini Kit, Westburg, Leusden, The Netherlands). DNA quantity and quality were determined spectrophotometrically (Nanodrop, Wilmington, USA), only including samples with an A260/230 ratio above 1.8. DNA from Kreatech (Megapool reference DNA female, EA-100F) was used as reference DNA. Labeling and hybridization, and data analysis was performed according to the methods described by Haan et al. [83] In short, after DNA isolation, labeling (Enzo Genomic DNA Labeling kit, Enzo Life Sciences, Raamsdonksveer, The Netherlands) and purification were undertaken (QIAGEN MinElute PCR Purification Kit, Westburg, Leusden, The Netherlands) with an elution volume of 2 × 10.5 μl, according to the manufacturer's instructions. Cy3- and Cy5-labelled DNA samples were combined with Cot-1 DNA (Invitrogen, Breda, The Netherlands) and blocking agent in hybridization buffer (Agilent Technologies).

### Hybridization

Hybridization on an Agilent SurePrint G3 Human CGH Microarray 4 × 180 K array the design of which can be found in the Gene Expression Omnibus (GEO) [84] platform GPL8687 (http://www.ncbi.nlm.nih.gov/geo) was performed for 24 h at 65°C. Microarrays were scanned using the Agilent Technologies Scanner G2505C (Agilent Technologies), and scans were quantified using the Agilent Feature Extraction software (version 10.5.1.1; Agilent Technologies, protocol CGH_105_Dec08) using default settings. The oligonucleotides were mapped along the genome according to the NCBI36/hg18 built (March 2006).

### DNA copy number data pre-processing

Genome data analysis was performed using R Version 2.6.2. The aCGH profiles were dewaved [85], and median was normalized. Segmentation was performed using the Bioconductor R-package DNAcopy version 1.22.1 [86] and subsequently DNA copy number calls for loss, normal, gain or amplification were made using the using R-package CGHcall version 2.8.0 [87]. Accuracy of normalization, segmentation and calling was verified by visual inspection using Nexus Copy Number (version 5, www.BioDiscovery.com). Raw and normalized data are online available on the GEO [84] platform GSE74633. (http://www.ncbi.nlm.nih.gov/geo/query/acc.cgi?acc=GSE74633).

### Gene expression microarray

### Sample preparation

Wild type CCRF-CEM cells were harvested for RNA isolation after 24 hours of incubation with 7 nM BTZ (CEM/WT_BTZ). The several BTZ resistant sublines were cultured without BTZ for 7 days, prior to a 24 hour exposure on the BTZ concentration on which they normally thrive (CEM/BTZ7 with 7 nM, CEM/BTZ100 with 100 nM and the CEM/BTZ200 with 200 nM of BTZ). Expression ratios were calculated using the untreated CEM/WT as a reference. CEM/BTZ-200, referring to CEM/BTZ200 cells cultured in absence of BTZ for several weeks, were included as an additional reference but were not exposed to BTZ prior to gene expression array experiments.

### RNA isolation and quality control

Total RNA was isolated using RNAbee (*AMSBiotechnology),* according to the protocol provided by *AMSBiotechnology*. Nanodrop ND-1000 was used for accurate nucleic acid concentration measurements. Quality control was performed using Agilent 2100 Bioanalyzer (Agilent Technologies) in combination with the Agilent RNA 6000 NanoLabChip kit to visualize and quantify the amount of RNA. Samples with a RNA Integrity Number (RIN) of > 7, concentration of > 5 μg/ml and two distinct peaks corresponding to the 28S and 18S ribosomal RNA bands at a ratio of 1.8–2.0 as seen on Agilent electrophorogram were used.

### Expression microarray

After RNA isolation, labeling (labeling performed with Agilent Low RNA Input Fluorescent Linear Amplification Kit, Agilent Technologies) and purification were undertaken (RNeasy Mini Kit, Agilent Technologies). Equal amounts of Cy3-CTP and Cy5-CTP labeled samples were hybridized to Agilent 4 × 44 K Whole Human Genome arrays (Agilent Technologies) the design of which can be found in the GEO [84] platform GPL4133 (http://www.ncbi.nlm.nih.gov/geo), according to the manufacturer's instructions. Hybridization was carried out for 17 h with rotation at 65°C in a hybridization oven. Microarrays were scanned using the Agilent DNA Microarray Scanner (Agilent Technologies), and scans were quantified using the Agilent Feature Extraction software (Agilent Technologies).

### Expression data pre-processing

Raw expression data generated by the Feature Extraction software were imported into the R statistical environment using the LIMMA package [88] in bioconductor (http://www.bioconductor.org). After background correction, the intensity distributions within and between arrays were normalized using the *Loess* [89] and *quantile* [90] algorithm, respectively. Raw and normalized data are online available on the GEO [84] platform GSE74634. (http://www.ncbi.nlm.nih.gov/geo/query/acc.cgi?acc=GSE74634).

### miRNA expression array

The miRNA expression profiles of the samples were profiled using Agilent human miRNA Microarray V2 (Agilent Technologies, Santa Clara, CA). Each array contained 60-mer probes representing 723 human and 76 human viral miRNAs from the miRBase (Version 10.1) the design of which can be found in the GEO [84] platform GPL8227 (http://www.ncbi.nlm.nih.gov/geo). The array experiment was carried out using Agilent miRNA system protocol v2.0. Briefly, each RNA sample was labeled with Cyanine3-pCp and hybridized to the Agilent human miRNA microarray using the miRNA Complete Labeling and Hyb Kit (Agilent p/n 5190–0456). The slide was washed using Gene Expression Wash Buffer kit (Agilent p/n 5188–5327), and then scanned using an Agilent DNA microarray scanner. The raw miRNA expression data were extracted from the scanned image using Agilent Feature Extraction Software V10. Coefficient of variation (CV) within groups of replicate probes was used as a quality control measure to reflect the intra-array reproducibility. The raw expression values of miRNA were imported into Agilent GeneSpring Software V10 for normalization and identification of differentially expressed miRNAs between bortezomib-resistant and wild-type cells.

### Data analysis

After normalization as described above, basic data handling and data preparation for further analysis was performed using Microsoft Excel 2010, clustering and subsequent visualization was performed using Cluster 3.0 and Treeview [91]. GO gene annotation was obtained from Database for Annotation, Visualization and Integrated Discovery (DAVID) [92], pathway analysis and gene set enrichment analysis were performed using GeneGoMetacore (http://www.genego.com/metacore.php) and Gene Set Enrichment Analysis (GSEA, Broad institute) [93, 94]. Integrated analyses of DNA copy number, gene expression microarray and miRNA expression data were performed as described by Menezes et al. [95] and Van Iterson et al. [96].Raw and normalized data are online available on the GEO [84] platform GSE74632. (http://www.ncbi.nlm.nih.gov/geo/query/acc.cgi?acc=GSE74632).

### Immunocytochemistry

Cytospins were fixed for 10 min with 4% of paraformaldehyde (PFA) in PBS pH 7.4, permeabilized for 10 min with 0.1% of Triton-X100 in PBS and blocked with 10% of fetal calf serum (FCS) in PBS for 1 hour. Primary (anti-MARCKS diluted 1:50, anti-ubiquitin 1:20) and secondary antibodies (diluted 1:100) were incubated for 1 hour each at 4°C. Nuclei were stained with 300 nM 4′,6-diamidino-2-phenylindole (DAPI). Images of the cell line experiments were obtained using the Zeiss Axiovert 200M Marianas™ inverted microscope connected to a cooled Cooke Sensicam SVGA CCD camera [Cooke Co., Tonawanda, NY] as previously described by Wojtuszkiewicz et al. [97]. DAPI, Alexa 488 and Alexa 647 were irradiated with a pulsed laser at 405 nM, 499 nm and 654 nm respectively. A 63× oil objective with NA 1.4 was used to image the sample. Data collected were processed using SlideBook™ software (SlideBook™ version 5.5.2.0 [Intelligent Imaging Innovations, Denver, CO]). The data acquisition protocol included optical planes to obtain 3-D definition.

Confocal scanning laser microscopy was performed on a Leica TCS SP8 STED 3X (Leica Microsystems) as previously described by Wojtuszkiewicz et al. [97]. Abberior STAR 580 and Alexa 647 were irradiated with a pulsed white light laser at 588 nm and 654 nm respectively. A 100× oil objective with NA 1.4 was used to image the sample. Detection of the fluorescent signal was performed with gated Hybrid Detectors. Finally, the images were deconvolved using Huygens Professional (Scientific Volume Imaging).

## SUPPLEMENTARY MATERIALS FIGURES AND TABLES


