# Supplementary material for: Exocytosis of polyubiquitinated proteins in bortezomib-resistant leukemia cells: a role for MARCKS in acquired resistance to proteasome inhibitors
Source: Oncotarget. 2016 Aug 17;7(46):74779–96. doi: 10.18632/oncotarget.11340 (PMC5342701; doi:10.18632/oncotarget.11340)
Supplement: Supplementary file 1 [file oncotarget-07-74779-s001.pdf]

Exocytosis of polyubiquitinated proteins in bortezomib-resistant leukemia cells: a role for MARCKS in acquired resistance to proteasome inhibitors

Supplementary Materials

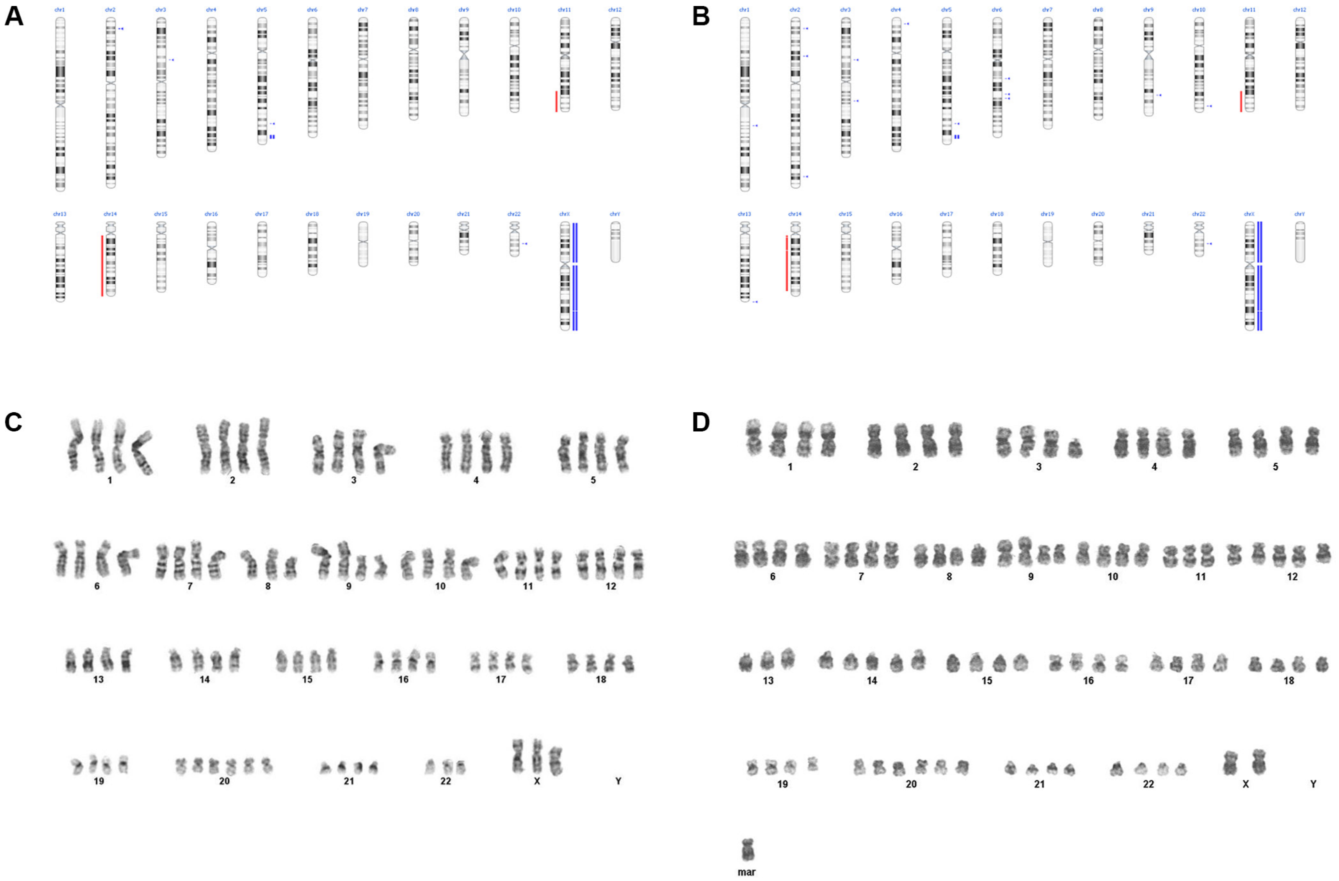

Supplementary Figure S1: ArrayCGH analysis of BTZ-resistant CEM cells as compared to CEM/WT. (**A**) CEM/BTZ7 vs CEM/WT, (**B**) CEM/BTZ200 vs CEM/WT. Red marks represents a gain of expression in the BTZ resistant CEM cells, blue a loss. Representative karyotype of CEM/WT (**C**) : 91,XXX,-X,-8,del(8)(p11.2),add(9)(p24)x2,del(9)(p22)x2,+20,+20,-22, and CEM/BTZ200 (**D**): 93,XX,-X,-X,del(3)(p11),del(8)(p11.2),?del(8)(p11.2),add(9)(p24)x2, del(9)(p22)x2,+14,+20,+20,+mar.

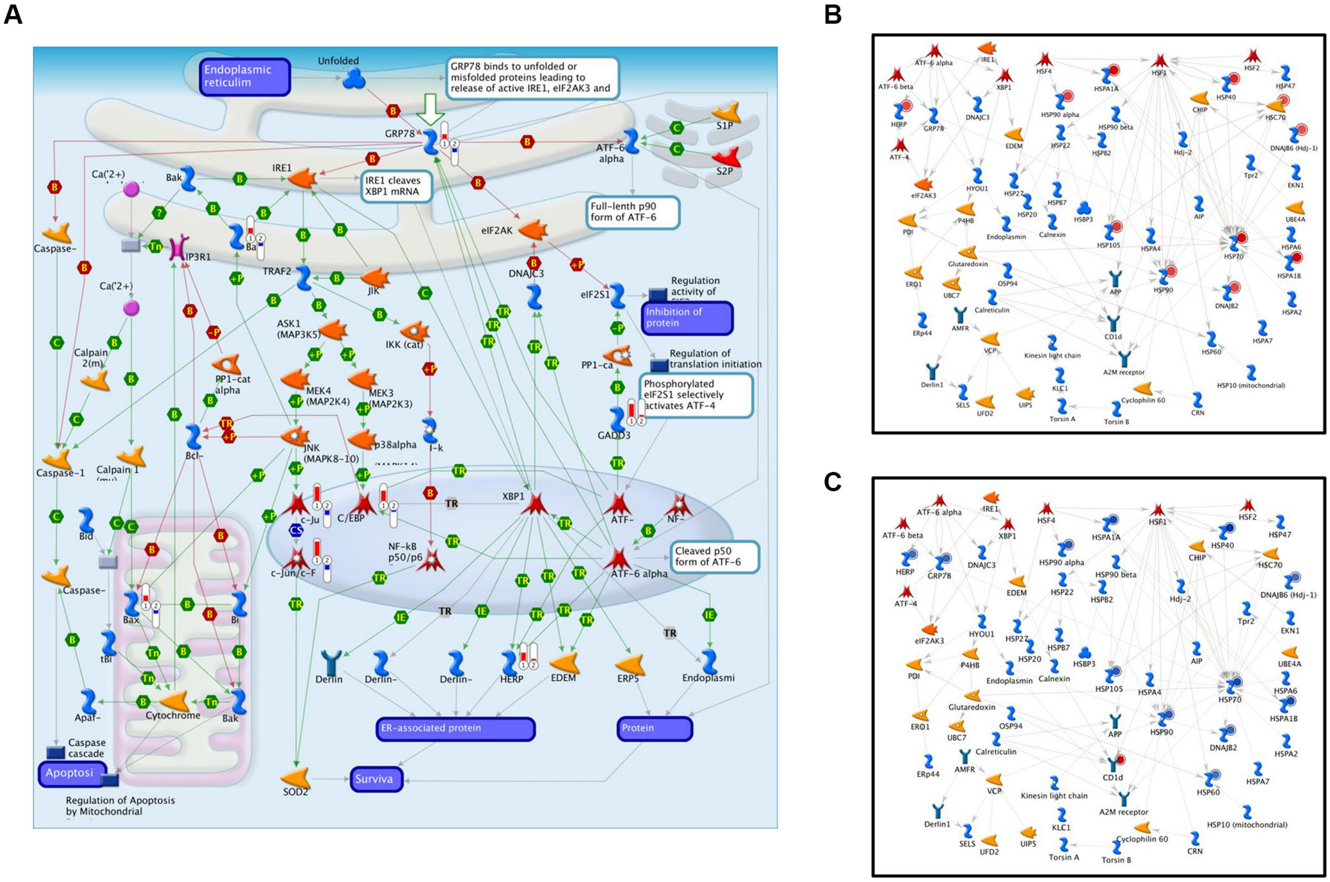

Supplementary Figure S2: Graphical representation of 2 highly affected pathways identified by pathway analysis of GEP using GeneGO software. Only genes with > Log2 fold change (CEM/WT_BTZ and CEM/BTZ7) in gene expression were used. Red indicates upregulation, blue downregulation. The arrows symbolize the different interaction between the proteins; green represents activation/induction, red inhibition / repression and gray unspecified. (**A**) The "Apoptosis and survival Endoplasmic stress response pathway", CEM/WT_BTZ vs CEM/WT (bars labeled 1) and CEM/BTZ7 vs CEM/WT (bars labeled 2). B = binding, C = cleavage, IE = influence on expression, −P / +P = de- and phosphorylation, T = transformation, Tn = transport and TR = transcription regulation. (Full legend can be found on http://lsresearch.thomsonreuters.com/static uploads/files/2014-05/MetaCoreQuick ReferenceGuide.pdf). "Protein folding response to unfolded protein response" network process, (**B**) ratio of CEM/BTZ vs CEM/WT and (**C**) CEM/BTZ7 vs CEM/BTZ.

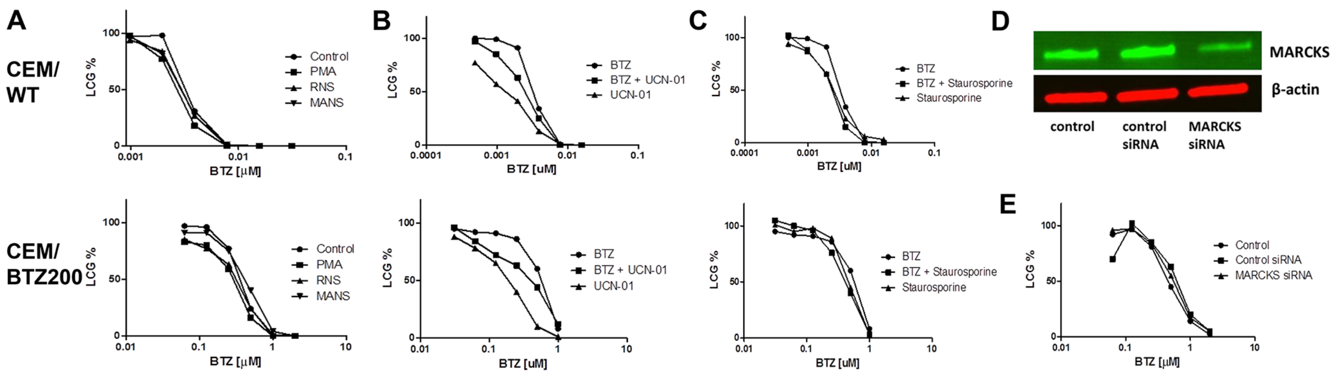

Supplementary Figure S3: BTZ dose response curves for (A) CEM/WT and CEM/BTZ200 cells before and after incubation with 50 nM PMA (stimulation phosphorylation of MARCKS), 100 uM MANS (MARCKS inhibition) or 100 uM RNS (MANS negative control) for 1 hour. Single and combined dose response curves of BTZ together with the PKC inhibitor UCN-01 (**B**) or staurosporine (**C**) in CEM/WT and CEM/BTZ200 cells. (**D**) MARCKS downregulation in CEM/BTZ200 cells after siRNA (Western blot). (**E**) BTZ dose response curve CEM/BTZ200 cells after siRNA MARCKS, including negative controls.

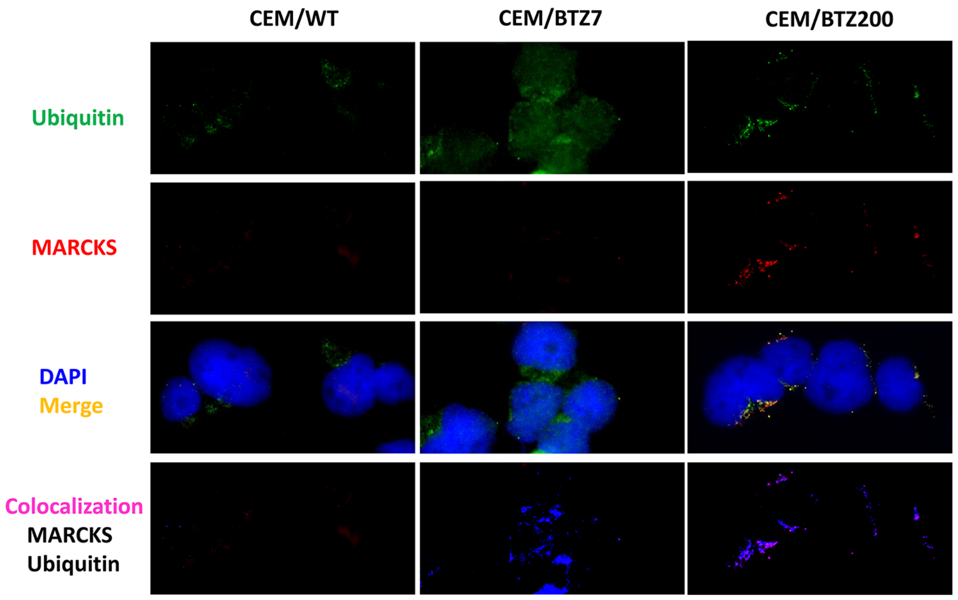

Supplementary Figure S4: MARCKS and ubiquitin protein expression in untreated CEM/WT cells, CEM/BTZ7 cells treated with 30 nM BTZ for 1 hour and CEM/BTZ200 cells treated with 400 nM BTZ for 1 hour. First row depicting ubiquitin single staining, second row MARCKS single staining, third row the combination of DAPI nuclear staining (Blue), ubiquitin (Green), and MARCKS (Red) and the last row shows the level of co-localization of MARCKS with ubiquitin as calculated by the SlideBook microscope software depicted in blue to magenta (strong co-localization).

Supplementary Table S1: Validated targets of differentially expressed miRNA's in CEM/BTZ100 and CEM/BTZ200 cells as compared to CEM/WT (Targets identified by MIRWALK)

miRNA Name	Log 2 Fold Change	Validated target genes
**hsa-mir-101**	2,51	AHSA1,AKT1,ALK,APP,ATP5B,ATP8A2,BBS9,BCL2L11,BCR,CACNA1C,CACNB1,CSN1S1,CSN2,CSN3,CTLA4,CYP2A6,DCP1A,DUSP1,EIF3A,EPHB2,EZH2,FOS,FOSB,FRAP1,FRAP1,HOXA1,ICOS,ITGA2B,MAFB,MAGI2,MAPK8,MCL1,NPC1,PIK3CA,PPIG,PRKCA,PTEN,PTGS2,RC3H1,RC3H2,TGFB1,TIA1,USF2,WNT5A,ZIC1
**hsa-mir-149**	−2,71	AATF,AKT1,ATF1,ATM,BAP1,BCL2,BRCA1,BRCA2,CDKN1A,CISH,COX8A,CSH1,CTSD,DICER1,E2F1,HOXB8,IGF1R,IGFBP5,IGFBP6,INS,KPNA2,MRE11A,MYBL2,NBN,PAWR,PCAF,PCNA,POLR2K,RB1,TP53
**hsa-mir-150**	−2,72	AKT1,ALG2,APC,ASAHL,BCL2,CAPNS1,CCND1,CD4,CD8A,CDK4,CDK6,CLCN3,CREB1,CTNNB1,CTSL1,E2F1,E2F3,EGR2,ELA2,FASN,FH,FOXP1,FZD7,GPI,HMGA2,IGF1,IL17A,IL6,IRAK2,IRAK4,JAK2,JUN,KIT,MAP3K8,MYB,NFKB1,P2RX7,PRKCA,PTGS1,RAB27A,RAC1,RB1,RB1,RELB,RHOU,SLC9A3R1,SOX17,SSSCA1,TCF7L2,TLE1,TNFSF10,TP53,WNT10A
**hsa-mir-181c**	3,52	AFP,AKT1,ATM,BAALC,BCL2,BCL2L11,BCR,CD36,CD4,CD8A,CD9,CDX2,CEBPA,DMPK,DNAH8,EPOR,ERBB2,ERG,FASTK,FLT3,GATA1,GATA6,GPI,GYPA,HOXA11,IL2,IL6,KRAS,LYN,MARK2,MCL1,MECP2,MMP9,MRGPRX3,MT4,NBN,NLK,NOTCH2,NOTCH4,NPM1,PML,PTEN,RASSF1,RC3H1,RDX,SMAD4,SOCS1,STAT1,TACSTD1,TCL1A,TFRC,TGFB1,TIMP3,TLR4,TNK2,TSPO,TWIST1,WT1,ZAP70,ZFPM1
**hsa-mir-19b-1**	2,06	ATXN1,CDKN1A,DDX20,EIF2C2,GEMIN4,GEMIN5,HIF1A,HIPK3,HNRNPA1,LAMC2,ME1,MYC,MYLIP,PIK3CA,PTEN,RNASEN,RNPC3,RRBP1,SETD2,SIP1,STMN1
**hsa-mir-210**	−3,48	ABCB9,ACVR1B,APC,ATP11C,BBS9,BDNF,BNIP3,CD40,CDH17,CDK10,CHD9,CLASP2,COX10,COX4I1,COX4I2,COX8A,CPEB2,DCT,DDAH1,DIRC2,DUSP9,E2F3,EFNA3,ELK3,ERBB4,FAM116A,FGFRL1,FOXJ2,GATA3,GPD1L,HECTD1,HIF1A,HOXA1,HOXA3,HPRT1,ISCU,ITLN1,KIAA1161,KLK3,KRT12,LONP1,MATR3,MDGA1,MIB1,MID1IP1,MNT,MYC,NCAM1,NIPBL,NPTX1,NR1H2,NT5E,P4HB,PECAM1,PIM1,PNCK,PNLIP,POMC,PPIA,PPM1D,PROC,PSAT1,PTPN1,RAD23B,RAD52,RIT2,ROS1,SCPEP1,SEH1L,SERTAD2,SETD2,SMCHD1,TBP,TIMM8A,TNPO1,TP53I11,UBQLN1,VEGFA
**hsa-mir-23a**	−2,62	ACTG1,AIFM1,AKT1S1,ALAS1,BDNF,C6orf134,CCND1,CCNT2,CD4,CD69,CDKN1A,CDKN2A,CDKN2D,CEBPA,CEBPB,CIB1,CSF1,CUGBP1,CUGBP2,CXCL12,DDIT3,DGCR8,DICER1,DLL1,DMTF1,E2F1,EIF2C1,EIF2C2,EZH2,FADD,FASN,FBXW11,FLI1,FOXP3,G6PD,GPD1,HDAC9,HES1,HIST1H2AE,HIST1H2BB,HMGA2,HMOX1,HOXA5,HSF1,ID3,IL2RA,IL6R,KIAA0152,KLF5,LAMC2,LITAF,LMNB1,LRPAP1,MAPK14,MECP2,MYC,NFATC3,PAK1,PARP8,PDGFB,PDK4,PLK2,POU4F2,PTEN,RING1,RNASEN,ROS1,RPE,RPIA,SELL,SMAD2,SMAD3,SMAD4,SMC1A,SPI1,SSSCA1,STK24,STK3,TAF9,TMEM184B,TMOD3,TPPP3,UBL4A,VAMP2,VEGFA,ZEB1
**hsa-mir-33b**	−2,11	ABCA1,BCL2,HMGA2,HSD11B1,SREBF1,SREBF2,TP53
**hsa-mir-595**	3,04	DICER1
**hsa-mir-625**	2,76	NTRK3
**hsa-mir-7-1**	2,47	ABCC1,AKT1,ARSF,BAX,BCL2L11,CBX5,CCND1,CCT4,CDC42,CDKN1A,DAP,DICER1,E2F3,EGF,EGFR,EPHB2,ERBB2,ERCC4,ERF,ETS2,FGFR3,HELLS,HOXD10,IGF1,IGF1R,IMPDH1,IRS1,IRS2,JAG1,MAEL,MCM2,MOCOS,MYC,NKX2

Supplementary Table S2A: Top10 gene sets enriched as determined by GSEA (Broad institute) for CEM/WT_BTZ vs CEM/WT

Gene Set	NES	Gene Set	NES
GERY CEBP TARGETS	2.60	CONCANNON APOPTOSIS BY EPOXOMICIN DN	−2.67
PODAR RESPONSE TO ADAPHOSTIN UP	2.55	LEE EARLY T LYMPHOCYTE UP	−2.65
KAN RESPONSE TO ARSENIC TRIOXIDE	2.48	GRAHAM NORMAL QUIESCENT VS NORMAL DIVIDING DN	−2.62
GARGALOVIC RESPONSE TO OXIDIZED PHOSPHOLIPIDS BLUE UP	2.45	MORI IMMATURE B LYMPHOCYTE DN	−2.61
NOJIMA SFRP2 TARGETS UP	2.45	GARGALOVIC RESPONSE TO OXIDIZED PHOSPHOLIPIDS TURQUOISE DN	−2.58
CONCANNON APOPTOSIS BY EPOXOMICIN UP	2.44	GRAHAM CML DIVIDING VS NORMAL QUIESCENT UP	−2.40
HOEBEKE LYMPHOID STEM CELL DN	2.44	HAHTOLA SEZARY SYNDROM DN	−2.36
HELLER SILENCED BY METHYLATION DN	2.43	ROSTY CERVICAL CANCER PROLIFERATION CLUSTER	−2.31
WOOD EBV EBNA1 TARGETS UP	2.39	REACTOME PURINE METABOLISM	−2.26
UEDA CENTRAL CLOCK	2.35	SCHLOSSER MYC TARGETS AND SERUM RESPONSE UP	−2.24

Detailed information about the identified gene sets can be obtained from (http://www.broadinstitute.org/gsea). NES = normalized enrichment score as provided by de GSEA program.

Supplementary Table S2B: Top10 gene sets enriched as determined by GSEA (Broad institute) for CEM/BTZ7 vs CEM/WT_BTZ

Gene Set	NES	Gene Set	NES
SCHUHMACHER MYC TARGETS UP	2.02	GARGALOVIC RESPONSE TO OXIDIZED PHOSPHOLIPIDS MAGENTA UP	−3.00
DAUER STAT3 TARGETS DN	1.94	ONDER CDH1 TARGETS 3 DN	−2.34
BILBAN B CLL LPL DN	1.93	SUH COEXPRESSED WITH ID1 AND ID2 UP	−2.12
SIMBULAN UV RESPONSE NORMAL DN	1.93	KRIGE AMINO ACID DEPRIVATION	−2.12
HANN RESISTANCE TO BCL2 INHIBITOR UP	1.90	PACHER TARGETS OF IGF1 AND IGF2 UP	−1.86
MENSSEN MYC TARGETS	1.89	FINETTI BREAST CANCERS KINOME GRAY	−1.70
TAKEDA TARGETS OF NUP98 HOXA9 FUSION 16D UP	1.89	JI METASTASIS REPRESSED BY STK11	−1.69
DELYS THYROID CANCER DN	1.88	CAFFAREL RESPONSE TO THC 8HR 5 UP	−1.65
TAKEDA TARGETS OF NUP98 HOXA9 FUSION 8D UP	1.87	NIKOLSKY BREAST CANCER 1Q21 AMPLICON	−1.62
PICCALUGA ANGIOIMMUNOBLASTIC LYMPHOMA UP	1.87	TOMIDA METASTASIS DN	−1.61

Detailed information about the identified gene sets can be obtained from (http://www.broadinstitute.org/gsea). NES = normalized enrichment score as provided by de GSEA program.
